# Maternal and Paternal Education on Italian Monolingual Toddlers’ Language Skills

**DOI:** 10.3390/brainsci14111078

**Published:** 2024-10-28

**Authors:** Allegra Cattani, Emre Celik

**Affiliations:** Department of Developmental and Social Psychology, Sapienza University of Rome, 00185 Rome, Italy; emre.celik@uniroma1.it

**Keywords:** parental education, language development, vocabulary comprehension, vocabulary production, Italian-monolingual children

## Abstract

Background. Language development in toddlers can be influenced by social interactions in environments and proximal contexts with mothers and fathers. We present the literature on mothers’ and fathers’ education level and socioeconomic status on the child’s language development; further evidence is needed in the Italian-speaking context. Aims. The study aims to confirm the role of mother and father education level on toddlers’ language skills assessed with direct and indirect measures. Methods and Procedures. Participants were 51 Italian-speaking children aged 33 to 41 months. Children were tested with a lexical test (PinG test) for comprehension and production of nouns and predicates and a morpho-syntactic test for grammar comprehension (PCGO). Parents of the children completed a demographic form and the Italian adaptation of the MacArthur-Bates CDI long version. Two series of one-way ANCOVAs were performed to study the role of mothers’ and fathers’ level of education on separate measures of their child’s language. Outcomes and Results. Findings suggest that in most families, mothers’ level of education is higher than fathers’ level of education. There was no significant difference between children of parents with low–middle level of education and children of parents with high level of education for the grammar comprehension tasks (PCGO) and indirect measure of vocabulary production (MacArthur-Bates CDI). However, both mothers’ and fathers’ level of education appears to be significant for the direct measurement of word production. Conclusions and Implications. This study provides new evidence for the role of mothers’ and fathers’ education on the development of word production in children aged 33 to 41 months, contributing to enriching the literature on the Italian context; it lays the groundwork for future research on the social and environmental factors that can affect language development.

## 1. Introduction

Early language development is associated with the subsequent acquisition of cognitive skills as well as with higher academic achievements and labor market success [1].

Many studies documented the parental role and the parental input, the family composition, the family socioeconomic status, and their effects on children’s school achievements [2,3]. The influence of mothers and fathers may be important on young children when they form early language and social skills that constitute later development [4,5,6]. Also, early language develops with social interactions in environments and proximal contexts with caregivers. Bronfenbrenner’s ecological systems theory [7] posits that the proximal processes are the primary mechanisms in child development. Thus, social interactions with caregivers are examples of proximal processes that are linked to early language development [8], such as a child’s language development being influenced within the context of relationships with mothers and fathers. However, although some previous research indicated an effect of the parental education level on the language development and vocabulary acquisition, not enough literature explores this thematic in relation to paternal education level.

### 1.1. Mothers’ and Fathers’ Education on the Child’s Language Development

Mothers’ and fathers’ education level is considered in the Hollingshead Index (1975) [9], which has been used to estimate the family socioeconomic status, and it consists of a calculation of four factors: parental education level, occupation status, sex, and marital status. The study by Richels, Johnson, Walden, and Conture [10] concludes that across all levels of SES, calculated according to Hollingshead’s Index [9], maternal education is the factor that contributed to a significant amount of the variance for the vocabulary and language scores for typically developing children in preschool age; specifically, for participants with families in the lowest socioeconomic status range (<25th), maternal education was significantly related to receptive vocabulary (PPVT; [11]), expressive vocabulary (EVT; [12]), and both receptive and expressive vocabulary (Test of Early Language Development—TELD; [13]) of children. However, for participants whose family SES was in the highest (>75th) quartile, neither maternal nor paternal education contributed significantly to the variance in the participants’ scores on the language tests performed (e.g., PPVT, EVT, or TELD-SLQ).

Moreover, Fernald, Marchman, and Weisleder [14] investigated the relation between SES, calculated according to the Hollinshead Index mentioned above [9], and the language processing skills and vocabulary between 18- and 24-month-old children, whose families were divided into two groups: lower and higher SES. However, almost all the mothers of families with higher SES had a high-level education (college degree or higher), while few mothers in the lower-SES group had completed college education. Meanwhile, participants’ language skills were assessed using the MacArthur-Bates CDI and the young children’s gaze patterns in response to speech (looking-while-listening procedure” LWL) [15]. In this study, a significantly larger vocabulary and a higher language processing efficiency were already found at 18 months and 24 months between infants from higher-SES families compared to lower-SES families.

In another study by Hoff and Tian [16], two different studies were conducted. A first longitudinal study took place in the U.S. on 63 children between 16 and 30 months; mother–child direct speech conversations were videotaped in two different times at 10 weeks of distance; the measure of maternal speech consisted in the number of different types of words and mean length of utterance, while the measure of child vocabulary was the number of word types produced in 90 utterances during the observation time. Children of mothers with high SES developed their language skills faster compared to low SES children, and this difference was mostly evident for two years; moreover, high SES mothers used a richer vocabulary and longer utterances in talking to their children than the mid-SES mothers; however, a series of hierarchical regression analyses established that SES accounted for only 5% of the variance in the vocabulary size the children used. In this study, maternal education affects their verbal style in the amount and quality of verbal talk to the children [17,18]. A second study was conducted in China on more than 600 children aged 24 to 47 months old. Demographic information and the Mandarin adaptation of MacArthur-Bates CDI were collected, and it was concluded that children with higher-educated mothers had larger vocabularies. At the same time, there was no significant difference in grammatical development associated with maternal education. The role of SES was investigated by Der Nederlanden et al. [19] on 539 Dutch-acquiring infants aged 8–13 months from mid to high SES level; two parental questionnaires were submitted; the first was a demographic information form, and the second was a section of the LENA developmental Snapshot [20] combined with the Brigance Parent-Child Interactions Scale [21]. This study found no evidence of an effect of parental education on the language development of infants.

Schwab and Lew-Williams [22] review the literature of the previous decades and assert that differences in the quantity and quality of language that children hear have been demonstrated across low-, mid-, and high-SES groups, but also that differences in input and learning also exist within SES groups. 

Recently, Frank, Braginsky, Yurovsky, and Marchman [23] examined the role of socio-economic factors on vocabulary size in toddlers acquiring different languages via the data stored in the “Wordbank” of the respective language adaptations of the MacArthur-Bates CDI (e.g., English, Spanish, Mandarin, Korean, Italian, French, Croatian, Danish, Russian, Latvian, Norwegian, Portuguese, Slovak, Swedish, and Turkish).

Examining the maternal education of eleven languages, the authors of [23] state that maternal education was used as a proxy for SES, following previous work suggesting that maternal education is strongly related to SES variation, especially early in development [24,25]. The relationship between maternal education and children’s vocabulary was variable across countries in the Wordbank database, with a contradictory role of maternal education on vocabulary comprehension rather than on vocabulary production. In fact, children with high levels of maternal education generally have a larger productive vocabulary but unexpectedly smaller comprehension vocabulary. Possibly, over-reporting of comprehension by low-SES parents could account for the different effects of maternal education that were observed in comprehension compared with production. For the American English databases, items’ comprehension was strongly affected by maternal education, compared to the other languages examined; interestingly, in different languages, the words more likely to be understood by children of highly educated mothers were often animal-related, which could be assumed were related to reading books about animals. Instead, words negatively linked to maternal education level were kinship terms, sweets, and money-related terms. Regarding vocabulary production, a certain kind of college advantage was found in nearly every dataset of every country examined. Across languages, animal vocabulary was again more prevalent for children of higher-educated mothers.

Consistently, among U.S. parents, the role of parenting is positively associated with a child’s expressive vocabulary. Morgan et al. [26] reported that among the 8650 24-month-old toddlers, female children from higher socioeconomic status households that experienced higher-quality parenting had larger spoken vocabularies, measured with a modified version of Mac-Arthur Bates-CDI developed specifically for the Early Childhood Longitudinal Study-Birth Cohort (ECLS-B) by the CDI Advisory Board.

While mothers’ education role is consistently examined, research on fathers’ role in child development is limited. It has been suggested that fathers with higher education levels may be more involved in caregiving and socialization (PICCI and FFS scales, [27]) and have more harmonious parenting interactions with young children than fathers with lower education levels [28,29,30,31].

Moreover, Cabrera et al. [32] revealed that American fathers with higher education levels were positively associated with children’s receptive skills at 36 months of age (tested with PPVT-III) and with letter–word identification [33] at 64 months of age, as well as with other cognitive domains (Bailey Scales of Infant Development and Leiter Revised Scale) and reasoning and math knowledge. Fathers who have more than a high school education have children performing better in all developmental domains, such as cognition, language, and social and emotional development, compared to children with fathers with lower education levels, indicating that fathers’ education is an important factor in early child language development. Similarly, during a triadic play setting with their 24-month-old child, the father’s language input (output, vocabulary, turn length, and wh-questions) was less than the mother’s language input. At age 36 months, parent level of education and the total quality of childcare were significant predictors of child language. However, Law and Roy [34], after conducting a review collecting data on more than 2000 participants using parental reports (MacArthur-Bates CDI), remarked that low education and sociocultural levels in the family do not emerge as risk factors for language production, and they explain little if any variance in children’s early productive vocabularies.

Finally, recent work reflecting changes in the structures of legislation and regulation in the society of Finland, where Finnish fathers have better possibilities to involve themselves in the care of young children, suggests that paternal occupational status and the presence at home have a role on a child’s language development. The social class of fathers and father–child activities significantly influenced language development [35]. Also, in a longitudinal cohort study that examined the growth of expressive vocabulary in 685 children between 13 and 24 months of age using the MacArthur-Bates CDI, fathers who do not work full time or take longer paternal leave are therefore more present in their child’s life, and those with high occupational status had children with larger gains in vocabulary [36].

Nonetheless, empirical studies have found significant differences in cognitive and language abilities between children raised in families with low socioeconomic status and their more advantaged peers [25,37,38,39,40,41] and in academic abilities. Sirin [42] carried out a meta-analysis of articles published between 1990 and 2000 on socioeconomic status in over 100,000 students and showed a medium to strong correlation between family socioeconomic status and academic achievements [42].

### 1.2. Aims of the Study

The previous literature uses parental education as an estimate of the socio-cultural and socio-economic status of the family [23,43,44,45]. As seen from the studies examined, family education and socioeconomic status are all relevant factors in children’s development, and parental education level is often the main and most relevant component of the SES. However, there is still a gap in assessing mothers’ and fathers’ level of education and their role in early language development in the Italian context.

Therefore, the aim of this study is to confirm and compare the effect of mothers’ and fathers’ level of education on toddlers’ language development in vocabulary and grammar comprehension and vocabulary production.

R.Q.: How do maternal and paternal education levels influence children’s language development within specific socioeconomic and cultural contexts, and what roles do these factors play in early language acquisition?

## 2. Materials and Methods

### 2.1. Participants

Fifty-one Italian monolingual-speaking children aged 33 to 41 months (50.9% females; Mage = 36.9 months; SDage = 2.08) took part in this study. The children and their families were selected by opportunity sampling via nurseries or a pediatrician. Firstly, nursery directors were contacted by email and phone calls; then, if requested, a meeting in loco with the nursery managers and with the parents of the participants was organized to answer any possible questions; and in primary care pediatricians’ networks, an email was sent to parents for recruitment purposes.

Testing took place in cities of Italy (Rome, Naples, Salerno, and Lecce) in a small, enclosed room with the door closed to minimize external distractions. The child was present in the room, with two examiners facilitating the assessment and a teacher who remained to ensure the child’s comfort and provide additional support if necessary, or a parent. The space was furnished with a small table and appropriately sized chairs to accommodate the child participants. The setup was designed to create a focused, child-friendly environment while allowing for accurate data collection. The sessions were video recorded.

Data were collected by three female Italian-speaking psychology and medicine graduates who attended a two-day training prior to the start of data collection. Exclusion criteria for participants were having any known hearing difficulties or developmental delays, prematurity at birth, and exposure to a second language or an international environment.

Parents of two children did not fill out the checklist, and one was excluded from being an outlier. Children who became fussy during an assessment or performed 3 SD below the mean of the group on the language score of a tool were checked for exclusion. Three children were excluded from PinG comprehension, and two were excluded from PinG production. No child was excluded from the PCGO grammar comprehension.

In the Italian education system, the mandatory school is up to 16 years old, while high school is completed at 18/19 years old on average. In our study, we consider both those who attended only mandatory school and those who graduated from high school as “low–middle level of education”.

In our sample, 66.6% of mothers obtained a university degree or higher, while 33.33% of the mothers have a low–middle level of education. Meanwhile, 52% of fathers obtained a university degree or higher, and 48% have a low–middle level of education. The level of education of one father is missing.

A third of children (30%) have both parents with a low–middle level of education; 52% have both parents who obtained a university degree or a higher level of education. Moreover, 2% of participants have a mother with a low–middle level of education and a father with a university degree or higher, whereas 18% of participants had mothers with a university degree and fathers with a low–middle level of education.

Moreover, 27 (52.9%) of participants had a sibling, of which 20 (39.2%) have older siblings.

### 2.2. Instruments

#### 2.2.1. Demographic Information

Parents filled out a demographic information form to gather information on the child’s age (in months), child gender, mother education level (low–middle level; high level), father education level (low–middle level; high level), occupation of both parents, nationality, place of birth, and family composition. Data about any health problems of the child was also registered.

#### 2.2.2. Parole in Gioco

The “Parole in Gioco” test [46] is a screening tool for assessing lexical comprehension and production levels in preschoolers from 19 to 37 months. The PinG test [46] is composed of four subtests of 20 items each: noun comprehension (NC), noun production (NP), predicates comprehension (PC), and predicate production (PP), plus 2 training items for each section. The items consist of colored picture cards depicting an object (e.g., camion [truck]) or a context defining an action (e.g., telefonare [phoning]), a descriptive word (e.g., corto [short]), or a locative word (e.g., vicino [near]). For each item, children are requested to indicate the target figure in the comprehension tests (e.g., Dov’è il gatto? [Where is the cat?] choosing between three options presented) or to name the target image in the production tests (e.g., Che cos’è questo? [What is this?] when one picture card is presented).

For the comprehension tasks, the answers are correct if the child points, touches, or grabs the correct picture card. Correct answers are rated 1, so the highest raw score for the collapsed comprehension nouns and predicates subtests is 40. For the production tasks, the answers are considered “Correct” if the child says the word (singular or plural), the verb (infinitive or conjugated), or the adjective (singular or plural) in the male or female form. Correct answers are rated as 1, so the highest raw score for the collapsed production nouns and predicates subtests is 40.

Regarding reliability, internal consistency was high, with a Cronbach’s alpha of 0.85 for the comprehension tasks and 0.9 for the production tasks calculated on our sample.

#### 2.2.3. Prova di Comprensione Grammaticale con Oggetti

The “Prova di Comprensione Grammaticale con Oggetti—PCGO” [47] is a grammar comprehension test with objects composed of 25 sentences, divided into two parts. Part one assesses the comprehension of nuclear sentences; it comprises a “pretest 1 argument”, where is verified the understanding of the target nouns and verbs that are included in the test, then a “Test 1 argument”, which is composed of 3 sentences, and a “pretest 2 arguments”, composed of 3 sentences, are performed, followed by a “test 2 irreversible arguments” with 3 sentences and a “test 2 reversible arguments” with 3 sentences. To conclude part one, a “pretest 3 arguments” is applied, followed by a “test 3 arguments” of 6 sentences. The maximum possible score for the nuclear sentences is 15. Part 2 assesses the comprehension of grammatical sentences, and it is composed of one sentence of “pretest pronouns”, 7 sentences of “test pronouns”, and three sentences, respectively, of “pretest passives” and “test passives”. The maximum possible score for the grammatical sentences is 10.

Children’s task is to reenact the sentences using the toys provided by the examiner. If the child performed the actions correctly the first time, the score was 1, if the child performed correctly on the second attempt, the score given was 0.5, otherwise it was null.

The “Prova di Comprensione Grammaticale con Oggetti”, in terms of reliability and internal consistency was high, with a Cronbach’s alpha of 0.73 for the nuclear sentences, a Cronbach’s alpha of 0.85 for grammar sentences, and a Cronbach’s alpha for a total score of 0.73 calculated on our sample.

#### 2.2.4. MacArthur-Bates CDI Italian Adaptation Words and Sentences Long Version Form

This measure uses parental reports to assess the production of Italian words and sentences. This parental report was produced [43] as an Italian adaptation of the MacArthur-Bates Communicative Development Inventories [48].

It is composed of a list of words containing 23 sections, with 674 total Italian words, including sounds, animals, toys, objects, places, clothes, body parts, food, vehicles, furniture, people, routine expressions, verbs and auxiliary verbs, adjectives, pronouns, adverbs, interrogatives, prepositions, articles, conjunctions; for the purposes of our study, it was considered the total number of words, the total number of nouns and social words (collapsing nouns, people and routine expressions), the total number of predicates (collapsing verbs, auxiliary verbs and adjectives) and the total number of grammar functors (collapsing adverbs, pronouns, prepositions, articles, interrogatives, conjunctions).

In terms of reliability, internal consistency was high, with a Cronbach’s alpha of 0.86 on the MacArthur-Bates CDI Italian adaptation Words and Sentences long version form calculated on our sample.

### 2.3. Ethical Approval, Informed Consent, and Data Protection

Ethical approval for the study was sought and granted by La Sapienza’s Ethics Committee, ensuring compliance with institutional and legal standards for research involving minors (Prot. n. 0001606 of 12 December 2022). Prior to participation, informed consent was obtained from the parents or legal guardians of all participants. They were provided with detailed information regarding the purpose of the research, the procedures involved, and their right to withdraw at any time.

Sensitive data (e.g., name and date of birth) were treated confidentially and anonymized with a given alphanumeric code. Data regarding the entire research group was used exclusively for research and scientific dissemination. Consequentially, after compiling the data, it was not possible to trace the identity of the individual participants in any way. All information collected in electronic format was stored on password-protected computers, and the paper versions were locked in the office of the first author.

### 2.4. Procedure

Children completed a series of lexical comprehension and production and grammar comprehension. Moreover, parents of 51 participants were handed in to fill out the PVB, a vocabulary size check list [43], the Italian adaptation of the MacArthur-Bates CDI, and return it after observing their child at home for no longer than 15 days.

## 3. Results

### 3.1. Descriptive Results

Data analyses were conducted using SPSS software version 29.0.2.0. The homogeneity of the sample was checked for gender with the mother’s level of education (*χ*^2^(1, *N* = 51) = 0.628, *p* = 0.428) and the father’s level of education (*χ*^2^(1, *N* = 50) = 0.0, *p* = 1); homogeneity of the sample was also checked for age groups (33–36 months, 37–41 months) with the mother’s level of education (χ^2^(1, *N* = 51) = 0.160, *p* = 0.689), and the father’s level of education (χ^2^(1, *N* = 50) = 0.063, *p* = 0.802).

Then, the mother’s level of education was checked against the father’s level of education, showing a significantly higher level of education for mothers compared to fathers (Pearson χ^2^(1, *N* = 50) = 19.731, *p* < 0.001). This result agrees with the literature that shows women have higher education on average than men [49].

Moreover, the mean ages in months and the gender of the sample are reported in Table 1.

### 3.2. Data Analysis

Analysis of covariance (ANCOVA) is a statistical method that integrates ANOVA and regression, enabling comparisons of group means while controlling for continuous covariates. ANCOVAs were used to compute the language development scores in children from low–middle vs. high levels of education of either maternal or paternal level of education, accounting for the age of the children (age factor expressed in a month as a covariate).

Two series of separate univariate ANCOVAs were performed to determine statistically significant differences between low–middle and high levels of maternal and paternal education on the child’s correct language performance, controlling for age in months. On a single ANCOVA performed, the dependent variable was one of the language child outputs (for “PinG”: total PinG word comprehension, nouns comprehension, and predicates comprehension; total PinG word production, noun production, and predicates production; for “PCGO”: total PCGO sentence comprehension, nuclear sentences, grammatical sentences; for “MB-CDI”: total MacArthur-Bates CDI word production, noun and social words, predicates, functors). Partial eta squares values are included to indicate the magnitude of the effect size of the level of education factor or age factor on the language score of the children. According to Cohen’s guidelines, *η*^2^ = 0.01 indicates a small effect; *η*^2^ = 0.06 indicates a medium effect; and *η*^2^ = 0.14 indicates a large effect.

Preliminary checks were made to assess the equality of variances across groups to evaluate whether the variance of scores in the groups is similar and to determine whether data follow a normal distribution. Levene’s test and normality checks were carried out, and the assumptions were met.

### 3.3. Effects of Maternal Education on Language Test Results

Table 2 presents the mean scores for all the language tasks as a function of the mother’s level of education, using age as a covariate. In the ANCOVAs performed for total PinG comprehension, comprehension of nouns, and comprehension of predicates, the mother’s level of education was not significant. For total PinG production and production of predicates, the mother’s education level was also not significant, while for production of nouns, the mother’s level of education was found significant (*F*(1,46) = 5.23, *p* = 0.027) with partial eta squared *η*^2^ = 0.10 for the mother’s level of education, indicating a medium effect size, after controlling for age expressed in months, which was also significant, *F* (1,46) = 6.23, *p* = 0.016, and *η*^2^ = 0.12, also indicating a medium effect size.

Meanwhile, the effect of the mother’s education was not significant for total PCGO scores, nuclear sentences, grammatical sentences, grammar comprehension test, nuclear sentences, grammatical sentences, MacArthur-Bates CDI total number of words, MacArthur-Bates CDI total number of nouns and social words, MacArthur-Bates CDI predicates, and MacArthur-Bates CDI grammar functors.

The covariate age was significant for the total MB-CDI word production *F*(1,45) = 3.94, *p* = 0.053, *η*^2^ = 0.08, and for MB-CDI nouns and social words *F*(1,45) = 4.62, *p* = 0.037, *η*^2^ = 0.09.

In sum, the performance of the children with mothers’ high education level was significantly higher on the PinG production of nouns than those with mothers’ low–middle education level.

### 3.4. Effect of Paternal Education on Language Test Results

Table 3 presents the mean scores for the language tasks as a function of father level of education, using age as a covariate. In the ANCOVAs for total PinG comprehension, comprehension of nouns, and comprehension of predicates, the father’s level of education was not significant. For total PinG production, the effect of father’s level of education was significant (*F*(1,45) = 5.39, *p* = 0.025), with a medium effect size *η*^2^ = 0.11, as well as the significant covariate age *F*(1,45) = 5.50, *p* = 0.023, *η*^2^ = 0.11. The effect of father’s level of education was primarily due to the significant effect of the PinG production of nouns (*F*(1,45) = 11.96, *p* = 0.001), with a large effect size of *η*^2^ = 0.21. The age as a covariate for the production of nouns was also significant (*F*(1,45) = 8.850, *p* = 0.005, *η*^2^ = 0.16).

However, the effect of the father’s education level was not significant for the production of predicates and for the total PCGO scores, nuclear sentences, grammatical sentences, MacArthur-Bates CDI total number of words, MacArthur-Bates CDI total number of nouns and social words, MacArthur-Bates CDI predicates, and MacArthur-Bates CDI functors.

Age as a covariate was significant on the MacArthur-Bates CDI total number of words (F(1,44) = 5.74, *p* = 0.021, *η*^2^ = 0.12), MacArthur-Bates CDI total number of nouns and social words (F(1,44) = 6.07, *p* = 0.018, *η*^2^ = 0.12), and MacArthur-Bates CDI predicates (F(1,44) = 5.24, *p* = 0.028, *η*^2^ = 0.11).

In sum, the performance on the PinG total production and, in particular, the production of nouns of the children with fathers’ high education level was significantly higher than those with fathers’ low–middle education level.

To take parental education into consideration, i.e., families with the highest level of education achieved by either mother or father, not only mothers or only fathers, a series of *t*-tests were applied to compare the mean scores of the children with at least one parent with a high level of education and the children with both parents with low–middle level of education on all the language measures considered (PinG comprehension and PinG production and their subtests, PCGO nuclear and grammar sentences, MacArthur Bates CDI total number of words, nouns and social words, predicates and functors). All t-tests were not significant, except for the PinG production of nouns, where it was found that children with the highest parental education produced more nouns than those with the low–middle level of education (*t*(47) = 2.026; *p* = 0.048).

## 4. Discussion

This study examined the contribution of mother’s and father’s level of education on Italian monolingual toddlers’ language skills, with direct and indirect measurements. First, the analysis confirms that mothers’ level of education is overall higher than fathers’ level of education, in accordance with the OECD statistics (2022) [50], which state that in most European countries, including Italy, younger women (aged 25–34) are more likely to have completed tertiary education than men in the same age group, reflecting global trends toward higher educational attainment for women. Secondly, mothers’ education level is analyzed in relation to the participants’ language skills. It is assessed that the effect of mothers’ high education level impacts positively on children’s production of nouns of the direct task compared to children of mothers with low–middle level education. We did not find significant maternal educational level differences in the other child’s language tasks examined. Thirdly, fathers’ high level of education positively impacts children’s nouns and general word production in direct tasks. Like mothers, the fathers’ education level does not influence most of the language tasks applied.

As mentioned above, the role of parental education level on language outcome is controversial. Fenson et al. [48] found that children growing up in higher SES families show wider vocabulary, measured with the MacArthur-Bates CDI [43].

According to Schwab and Lew-Williams [22], the family’s socio-economic status can predict the language input children receive from their parents, affecting the language outcomes. Previous research considered parental education as a factor contributing to the SES [23,43,44,45]. Therefore, it was hypothesized that the maternal and paternal education levels, investigated separately, could predict children’s language development.

Frank et al. [23] explored the role of maternal education on language outcomes such as comprehension and production of words. According to their study, maternal education did not affect word comprehension; however, it was positively related to word production, estimated with indirect measurements in different languages such as MacArthur-Bates CDI.

In accordance with the literature, our study also found an effect of maternal education on noun production (Parole IN Gioco, [46]). However, we did not find any effect of maternal and paternal education on indirect measurements, which are parental reports, and therefore susceptible to parental interpretation.

Differently from the previous literature, in this study, paternal education is considered and analyzed separately from maternal education to explore the role of each parental figure on early language development.

A Finnish study suggests that with the implementation of new legislation, which allows Finnish fathers to have better possibilities to involve themselves in the care of their children, the fathers’ presence at home and the amount of father–child activities significantly influenced language development [35]. The concept of the father’s role is evolving, with a higher involvement of fathers in the child’s care; however, in the Italian context, the father’s role is changing slowly and is still locked in traditional roles, in which usually fathers’ rarely take the time to look after their children, with the exception of higher educated fathers who tend to be more involved or those who work from home.

Pancsofar et al. [51] suggested that fathers produced significantly less verbal output than mothers in a family free play situation; however, it appeared in this triadic interaction that mothers and fathers differed on the quantity of output but not on the quality of their output. In this study, the role of fathers’ education on language development is present but limited to the direct measurements of the production tasks.

Meanwhile, age, which was our covariate, is significantly related to “total production tasks”, “noun production task”, and the “MacArthur-Bates CDI checklist for words, nouns, and predicates.

A key limitation of this study is that the limited number of parents with low educational levels restricts the ability to examine the full spectrum of parental education’s impact on language development. As a result, the findings may primarily reflect the experiences of children from families with middle to high education levels, reducing applicability to those from lower educational backgrounds. It is relevant, therefore, to take into account the numerosity and the composition of our sample, which is composed of the majority of the cases (52%) by families with both parents with high level of education, a third (30%) of families with both parents from low–middle education levels, only one family with a high father education level and mother with low education level, and nine families with high mother level of education and fathers with low–middle level of education.

Given the characteristics of the sample, the group of children with the mother’s education and the group of children with parental education were similar, limiting the comparisons of children’s language between maternal and the level of family education. Furthermore, despite efforts to control potential unmeasured confounders, such as the quality of language spoken at home and the quality of care, including parental involvement and time spent at home, they may still influence the results.

The findings of this study highlight the importance of further investigating the role of parental education level on children’s language outcomes. Given the potential influence of maternal and paternal education on language development, future research should aim to include a more diverse range of educational backgrounds, particularly low educational levels, to provide a more comprehensive understanding of this relationship.

## 5. Conclusions

This study demonstrates the distinct influence of maternal and paternal education levels on children’s language development. Maternal education was found to be significantly associated with the child’s ability to produce nouns, while paternal education had a broader impact, affecting both noun production and general word production. However, for maternal and paternal education levels, no significant effects were observed on other language measures, including word comprehension, grammar comprehension, and the indirect measurement of vocabulary (assessed by the MacArthur-Bates CDI).

These findings suggest that while parental education shapes specific aspects of language production, its influence may be more limited in other areas of language development, such as lexical comprehension and grammatical understanding. The lack of significant effects in these areas underscores the complexity of language acquisition and suggests that additional factors beyond parental education may contribute to children’s broader language competencies. Future research should aim to explore these other contributing factors and further examine the nuanced roles of maternal and paternal education in diverse language outcomes.

## Figures and Tables

**Table 1:** Distribution of age and gender among the children.

	N	Mage	SD	% Females
Total PinG word comprehension	48	37	2.06	50
Nouns	48	37	2.06	50
Predicates	48	37	2.06	50
Total PinG word production	49	37	2.04	51
Nouns	49	37	2.04	51
Predicates	49	37	2.04	51
Total PCGO sentence comprehension	51	36.9	2.08	50.9
Nuclear	51	36.9	2.08	50.9
Grammatical	51	36.9	2.08	50.9
Total MB-CDI word production	48	36.9	2.09	52.1
Nouns and social words	48	36.9	2.09	52.1
Predicates	48	36.9	2.09	52.1

**Table 2:** Mother’s level of education on language tasks.

	Low–Middle Level of Education		High Level of Education		*N*	*F*	*p*	*η* ^2^
	*M*	*SD*	*M*	*SD*				
Total PinG word comprehension	35.88	3.18	36.74	2.07	48	1.26	0.267	0.03
Nouns	18.88	1.45	19.16	0.86	48	0.72	0.401	0.02
Predicates	17.00	1.97	17.58	1.80	48	1.05	0.311	0.02
Total PinG word production	25.12	4.66	27.81	6.06	49	2.69	0.108	0.05
Nouns	12.35	2.57	14.38	3.35	49	5.23	0.027 *	0.10
Predicates	12.76	2.54	13.44	3.27	49	0.54	0.464	0.01
Total PCGO sentence comprehension	17.65	3.84	17.60	3.30	51	0.00	0.986	0.00
Nuclear	12.64	1.87	12.69	1.88	51	0.02	0.892	0.00
Grammatical	5.00	2.26	5.00	2.26	51	0.01	0.920	0.00
Total MB-CDI word production	515.07	157.35	546.39	131.92	48	0.49	0.484	0.01
Nouns and social words	318.40	86.05	324.06	73.91	48	0.04	0.839	0.00
Predicates	133.40	51.94	149.97	39.82	48	1.47	0.232	0.03
Functors	63.27	26.29	72.36	20.77	48	1.64	0.207	0.03

* *p* < 0.05

**Table 3:** Father’s level of education on language tasks.

	Low–Middle Level of Education		High Level of Education		*N*	*F*	*p*	*η* ^2^
	*M*	*SD*	*M*	*SD*				
Total PinG word comprehension	36.22	3.00	36.71	2.03	47	0.41	0.524	0.01
Nouns	18.96	1.33	19.13	0.85	47	0.23	0.632	0.00
Predicates	17.26	1.94	17.58	1.79	47	0.36	0.554	0.01
Total PinG word production	25.17	4.33	28.76	6.25	48	5.39	0.025 *	0.11
Nouns	12.30	2.48	15.08	3.28	48	1.96	0.001 **	0.21
Predicates	12.87	2.44	13.68	3.44	48	0.81	0.374	0.02
Total PCGO sentence comprehension	17.40	3.69	17.90	3.32	50	0.16	0.688	0.00
Nuclear	12.59	1.93	12.81	1.86	50	0.10	0.753	0.00
Grammatical	4.81	2.04	5.1	1.87	50	0.17	0.681	0.00
Total MB-CDI word production	521.91	160.17	557.84	114.69	47	0.44	0.509	0.01
Nouns and social words	318.45	88.05	328.92	67.07	47	0.04	0.835	0.00
Predicates	138.50	52.41	153.40	32.30	47	0.96	0.332	0.02
Functors	64.95	25.42	75.52	17.03	47	2.31	0.136	0.05

* *p* < 0.05, ** *p* < 0.01.

## Data Availability

The authors confirm that the data supporting the findings of this study are available within the article. Raw data are available from the corresponding author upon request.
